# A Secure and Lightweight Fine-Grained Data Sharing Scheme for Mobile Cloud Computing

**DOI:** 10.3390/s20174720

**Published:** 2020-08-21

**Authors:** Haifeng Li, Caihui Lan, Xingbing Fu, Caifen Wang, Fagen Li, He Guo

**Affiliations:** 1 School of Software, Dalian University of Technology, Dalian 116024, China; lihaifeng8848@mail.dlut.edu.cn (H.L.); guohe@dlut.edu.cn (H.G.); 2 School of Electronic and Information Engineering, Lanzhou City University, Lanzhou 730070, China; 3 Guangxi Key Laboratory of Cryptography and Information Security, Guilin 541004, China; uestcfuxb@126.com; 4 School of Cyberspace, Hangzhou Dianzi University, Hangzhou 310018, China; 5 Guangdong Provincial Key Laboratory of Information Security Technology, Guangzhou 510275, China; 6 College of Big Data and Internet, Shenzhen Technology University, Shenzhen 518118, China; wangcaifen@sztu.edu.cn; 7 School of Computer Science and Engineering, University of Electronic Science and Technology of China, Chengdu 611731, China; fagenli@uestc.edu.cn

**Keywords:** attribute-based encryption (ABE), mobile cloud computing (MCC), verifiability, outsourced decryption, CCA-secure

## Abstract

With the explosion of various mobile devices and the tremendous advancement in cloud computing technology, mobile devices have been seamlessly integrated with the premium powerful cloud computing known as an innovation paradigm named Mobile Cloud Computing (MCC) to facilitate the mobile users in storing, computing and sharing their data with others. Meanwhile, Attribute Based Encryption (ABE) has been envisioned as one of the most promising cryptographic primitives for providing secure and flexible fine-grained “one to many” access control, particularly in large scale distributed system with unknown participators. However, most existing ABE schemes are not suitable for MCC because they involve expensive pairing operations which pose a formidable challenge for resource-constrained mobile devices, thus greatly delaying the widespread popularity of MCC. To this end, in this paper, we propose a secure and lightweight fine-grained data sharing scheme (SLFG-DSS) for a mobile cloud computing scenario to outsource the majority of time-consuming operations from the resource-constrained mobile devices to the resource-rich cloud servers. Different from the current schemes, our novel scheme can enjoy the following promising merits simultaneously: (1) Supporting verifiable outsourced decryption, i.e., the mobile user can ensure the validity of the transformed ciphertext returned from the cloud server; (2) resisting decryption key exposure, i.e., our proposed scheme can outsource decryption for intensive computing tasks during the decryption phase without revealing the user’s data or decryption key; (3) achieving a CCA security level; thus, our novel scheme can be applied to the scenarios with higher security level requirement. The concrete security proof and performance analysis illustrate that our novel scheme is proven secure and suitable for the mobile cloud computing environment.

## 1. Introduction

With the tremendous development of distributed computing technology and virtualization technology, cloud computing has gained popularity in various fields such as scientific research, economic finance, medical treatment, education and entertainment. In order to relieve the local storage burden and enjoy the great benefits provided by cloud computing, such as powerful computing power and conveniently ubiquitous access, more and more individuals and organizations are willing to outsource their local data to the remote cloud server for storing, maintaining, manipulating and sharing with others [1]. Meanwhile, as the modern electronic technique and wireless communication technology have gained impressive progress in the past years, mobile devices (e.g., smart mobile, tablet, smart sensor, PDAs) as convenient handheld communication tools have become increasingly popular and more promising than ever before due to their mobility, convenience and availability. With the popularization of various mobile applications, all kinds of mobile devices have been observed in various domains, such as mobile commerce [2], mobile learning [3], mobile health monitoring [4], and so on. It is well recognized that, although mobile devices can provide conveniently handheld ubiquitous access anytime and anywhere, they are also restrained by their relatively weaker computing power, lower battery power and smaller storage space. The above-mentioned weaknesses of mobile devices greatly hinder the realistic application with respect to the applications of intensive computational tasks and massive storage demands. Nevertheless, cloud computing paradigm can offer an unimaginable infinite storage space and tremendous computing resource. Thus, a natural idea is combining the merits of mobile devices and the advantage of cloud computing to create a new paradigm, whereby the cloud computing is responsible for performing the heavy computing-intensive tasks and storing massive data of mobile user’s as well as preserving the all the merits of mobile devices. Fortunately, in recent years, a seamless convergence has been observed between cloud computing and mobile device in various aspects and known as Mobile Cloud Computing (MCC). While the single widely accepted clear definition of MCC has still remained disputed and different scholar researches give different definitions of MCC from different prospective [5,6,7], based on the results of the research [8,9,10], in this work, we mainly refer to MCC as the well accepted fact that MCC can facilitate mobile users with the complicated data computing and unlimited storage services in the cloud server, thus, the mobile devices do not necessarily to equip with powerful configuration (such as, high CPU speed and large memory capacity) without sacrificing any desirable properties of the mobile devices. In short, mobile devices can seamlessly integrate with the premium powerful cloud computing.

Meanwhile, attribute based encryption has been envisioned as one of the most promising cryptographic primitives for providing secure and flexible fine-grained “one to many” access control, particularly in large scale distributed system with unknown participators. However, most of existing ABE schemes are not suitable for MCC because they involve expensive pairing operations which pose a formidable challenge for the resource-constrained mobile devices, thus, greatly delaying the widespread popularity of MCC.

In addition, due to size-limitation, it is impractical to solely depend on improving the hardware technique level to design top premium mobile devices with unlimited storage and computational power as same as the personal computer (PC). Therefore, it is essential to devise external devices or resources with partial or large support for the resource-constrained mobile devices to perform computationally intensive task. Fortunately, the emergence of mobile cloud computing can meet the demand of computation and storage resource for mobile device. The MCC is the integration of cloud computing and mobile computing, which can offer rich storage and computational resources over wireless networks to mobile users. In mobile cloud computing architecture, the heavy computation task and the massive data which have been previously done inside the mobile devices are being outsourced to the remote cloud server, accordingly, the physical control over the data of mobile user has been deprived. Thus, the mobile user may worry about whether their data is secure and privacy of outsourced sensitive data can be well preserved. User’s concerns about the security and privacy of their outsourced data are the primary barriers that hinder mobile cloud computing from widespread adoption by enormous potential mobile users to a large extent.

### 1.1. Related Work

During the past decades, the cryptographic researchers have developed many new cryptographic primitives. Among them, ABE is envisioned as one of the most attractive cryptographic primitive since it can provide secure and flexible fine-grained “one to many” access control, especially in large scale distributed system with unknown participators. The ABE is derived from the identity-based encryption mechanism [11], in which the identity information can be uniquely identified as the public key for encryption. In 2005, Sahai and Waters [12] first designed an identity encryption scheme based on biological characteristic information, called fuzzy identity-based encryption scheme (Fuzzy-IBE). The Fuzzy-IBE scheme can be regarded as the “prototype” of ABE. In 2006, Goyal, Sahai and Waters et al. [13] introduced the concept of attributes, and expanded fuzzy identity-based encryption to ABE. In their scheme, the user identity information is generalized to attributes relevant to user identity, and the private key and ciphertext are associated with a set of attributes, and the user will be able to decrypt the ciphertext if and only if the ciphertext attributes and the secret key attributes match each other to certain threshold. According to how the secret key and ciphertext are embedded with the access policy, the ABE-based schemes can be mainly divided into two types: Key-Policy Attribute-Based Encryption (KP-ABE) scheme [13] and Ciphertext-Policy Attribute-Based Encryption (CP-ABE) scheme [14]. For KP-ABE scheme, the user’s private keys are integrated with an access policy and the encrypted data are associated with a set of attributes. However, for CP-ABE scheme, the roles of an attribute set and an access structure switch to the opposite side. That is, in CP-ABE scheme, user’s secret key is embedded with a set of attributes and encrypted data are associated with an access structure. By taking the advantages of ABE, many scholar researchers and industrial engineers have devised a number of novel schemes for securely sharing data in distributed systems such as cloud computing [15,16,17,18,19,20,21,22,23,24].

Despite the ABE primitive is very powerful and promising, it still suffers from an efficiency weakness due to the fact that the traditional ABE based schemes involve many expensive pairing operations. It would become a significant challenge for mobile users since their local resources are limited, especially in battery life, storage capacity and computing power. In order to handle the efficiency problem of the local ABE schemes, in USENIX 2011, Green et al. [25] proposed an ABE based scheme with outsourced decryption by introducing an external cloud server (i.e., a proxy server) and using a transformation key from the users to significantly simplify the ciphertext in the cloud server side. In their new paradigm, the cloud server performed the majority expensive pairing operations while only left a small amount of lightweight computation task for local users, thus, greatly relieving the computation cost of ABE in the user side. However, the creative work of Green et al. [25] can accelerate the decryption procedure for local users by using untrusted servers, which may bring about a new security loophole that is how to guarantee the correctness of the transformation ciphertext since the aided decryption server is not fully trust. The semi-trust cloud server maybe deliberately return a false transformed ciphertext to the local user for saving its resource to gain more profits or other reasons, which will result in the user obtains the incorrect decryption results. In order to ensure the correctness for the transformed ciphertext, Lai et al. [26] developed an outsourcing attribute-based encryption with checkability to check the correctness of the transformed ciphertext returned by the untrusted cloud server, which can not only offload the heavy intensive computing tasks for local users, but also guarantee the correctness of the outsourcing decryption. It is no doubt that checkability is tremendous progress for outsourcing ABE schemes. Subsequently, many ABE based outsourcing schemes with various security properties have been proposed so far [27,28,29,30,31,32,33,34,35,36,37].

### 1.2. Motivation and Contribution

In order to attract the considerable potential mobile users not hesitate to adopt the mobile cloud computing any longer, the cloud service provider should tackle these security and privacy issues to provide a completely secure and ease environment. Apparently, to eliminate the user’s concerns on their outsourced data, a natural method is to encrypt the sensitive data before outsourcing them to the cloud servers; however, the encrypted data will bring about new issues. For instance, retrieval or sharing the encrypted data is greatly difficult. Traditional access control techniques are almost for plaintext, while in the mobile cloud computing environment, the privacy data and sensitive data of mobile user are stored in the cloud server in the form of ciphertext. Therefore, in this circumstance, the distributed access control technique over ciphertext plays a key role. In general, there are two non-interactive access control techniques adopting in the cloud computing. One is Fully Homomorphic Encryption (FHE) [38,39,40]. The proposals [41,42,43] adopt the fully homomorphic encryption technique, which can fully support the addition, and multiplication homomorphic operations and can be directly manipulated over the encrypted data without revealing any sensitive information. It seems an ideal access control technique especially suitable for the cloud computing setting. However, this technique is merely a theoretical approach and is currently inefficient and impractical in real-world application because this solution is required a huge expensive computational overheads. The other distributed access control technique over ciphertext is based on ABE. ABE mechanism is a secure and flexible fine-grained access control, especially applicable for large scale distributed system with unknown participators. While CP-ABE scheme can provide fine-grained access control and is applicable for cloud computing, adopting a CP-ABE scheme directly into a mobile cloud computing that may yield some open issues. One of the most severe drawbacks of the current ABE schemes is that the inherent low efficiency problem, which becomes more severely in the storage limited and computation resource-constrained mobile devices. For example, most current ABE schemes mainly adopt the bilinear maps which will bring about huge expensive pairing operations of ABE schemes. Moreover, the high computation costs during decryption procedure grows linearly along with the number of attributes involved in the access policy. It is a serious challenge for resource-limited mobile users to perform these time-consuming pairing operations; and thus, creating a bottleneck for efficiency of mobile cloud computing. This will greatly impede the widespread adoption of mobile cloud computing. As a response to this problem, many cryptographic scholars have constructed multiple attribute-based encryption with outsourced decryption schemes to reduce the heavy computation costs in mobile devices, such as [25,26,29,44]. While these outsourced attribute-based encryption works have made great process to improve the efficiency of mobile cloud computing, the bottleneck of the efficiency of mobile cloud computing is not fully addressed. For instance, scheme [25] only supports outsourcing the expensive pairing operations to the cloud server, but it does not consider whether the cloud sever returns the corrected transformed ciphertext. It is essential to ensure the validity of the transformed ciphertext from the cloud server because it is untrusted entity and it may cheat the user with forged ciphertext for some evil purpose or just for economic benefits. Therefore, it is necessary to check the validity of the returned results from the cloud server. Later, some scholars investigated the checkability on the returned outsourcing computation results and designed verifiable outsourcing decryption schemes, such as [26]. However, these schemes only support the chosen-plaintext attack(CPA) security level which will limits their application to some extent, and cannot be used in environment for the higher demand for security. As the mobile cloud computing gain an increasing popularity, it is of most urgent to address this realistic problem for improving the performance of the mobile cloud computing. In this work, by adopting the transformation key technique [25], we propose a secure and lightweight fine-grained data sharing scheme for mobile cloud computing scenario, which can provide verifiable outsourcing decryption for intensive computing task during decryption phase to the cloud server without revealing the user’s data or decryption key. Moreover, our proposal can achieve security against the chosen-ciphertext attacks (CCA) and thus can be used to the circumstance with higher security level requirements, such as medical data sharing. For instance, a doctor can access and diagnose the Personal Health Record (PHR) of the patients with a mobile device (such as mobile phone, tablet) conveniently by outsourcing the heavy computation operations to the MCC.

### 1.3. Paper Organization

The remainder of the paper is organized as follows. In Section 2, we discuss some relevant preliminaries used in our paper, such as bilinear pairing, complexity assumption, access tree. In Section 3, we present the problem statement, including the system model, the definition of our novel scheme and the security model. In Section 4, we present the detailed construction of our new scheme. In Section 5, we give the security analysis under the random oracle model. In Section 6, we conduct the concrete performance evaluation and compare the efficiency with other state-of-the-art schemes in terms of functionality, theoretical analysis and experimental simulation. Finally, we draw the conclusion of the whole paper in Section 7.

## 2. Preliminaries

### 2.1. Bilinear Pairing

**Definition** **1** (Bilinear Pairing). *Let $G_{1}$ and $G_{T}$ be two multiplicative cyclic groups with the equal prime order p and assume g is a generator of $G_{1}$, the map $e:G_{1}\times G_{1}\to G_{T}$ is defined as a bilinear map if and only if the following three properties are hold.*

*(1)*  *Bilinearity. For all elements $g_{1},g_{2}\in G_{1}$,$e\left({g_{1}}^{a},{g_{2}}^{b}\right)=e{\left(g_{1},g_{2}\right)}^{ab}$, where $a,b\in Z_{p}$ are two random numbers and $Z_{p}$ is a finite field.*
*(2)*  *Non-degeneracy. There exists elements $g_{1},g_{2}\in G_{1}$ such that $e\left(g_{1},g_{2}\right)\neq 1_{G_{T}}$, where 1 is the identity element of $G_{T}$.*
*(3)*  *Computability. For all elements $g_{1},g_{2}\in G_{1}$, there exists an efficient algorithm to compute $e(g_{1},g_{2})$.*

### 2.2. Complex Assumption

**Definition** **2** (DBDH Problem). *The Decision Bilinear Diffie–Hellman (DBDH) problem is defined as follows: Given $G_{1}$ and $G_{T}$ be two multiplicative cyclic groups with the equal prime order p, g is a generator of $G_{1}$, $e:G_{1}\times G_{1}\to G_{T}$ is a bilinear map, $Z_{p}$ is a finite field, consider the two following probability distributions: $D_{1}=D\left(g,g^{a},g^{b},g^{c},e{\left(g,g\right)}^{abc}\right)$, where a,b,c are randomly chosen in $Z_{p}$; and $D_{2}=D\left(g,g^{a},g^{b},g^{c},R\right)$, where a,b,c are randomly chosen in $Z_{p}$, and R is randomly chosen in $G_{T}$, there is no Probabilistic Polynomial Time(PPT) algorithm can distinguish the two probability distributions: $D_{1}$ and $D_{2}$ with non-negligible probability so far.*

*More formally, the advantage of a distinguisher against the DBDH assumption is defined to be:*

$$Adv\left(D\right)=|P_{a,b,c\in_{R}Z_{p},R\in_{R}G_{T}}\left[1\leftarrow D_{2}\right]-P_{a,b,c\in_{R}Z_{p}}\left[1\leftarrow D_{1}\right]|.$$

### 2.3. Access Tree

An access tree is used to describe an access structure. To facilitate the description, we define some notations as follows.

*T*: This represents an access tree representing the access structure.

*x*: This represents a node in the access tree *T*, which can be categorized into two types: Leaf node and non-leaf node (interior node). Each non-leaf interior node is represented a threshold gate, such as “AND” or “OR” threshold gate while each leaf node is associated with an attribute.

$num_{x}$: This represents the number of children of the node *x*.

$k_{x}$: This represents the threshold value of node *x*, where $0\leq k_{x}\leq num_{x}$. If $k_{x}=1$ and *x* is an interior node, it means that the threshold is an “OR” gate. If $k_{x}=num_{x}$ and *x* is an interior node, it means that the threshold is an “AND” gate. In particular, the threshold value of each leaf node *x* is defined as $k_{x}=1$.

parent(x): The function parent(x) is used to return the parent of the node *x* in the access tree.

index(x): The function index(x) is used to return a unique number associated with the node *x*, where the number is uniquely assigned to *x* in a certain manner.

att(x): The function att(x) is used to return an attribute associated with the leaf node *x* in the access tree.

$T_{x}$: This represents the sub-tree for *T* rooted at the node *x* in the access tree.

If an attribute set *S* matches the sub-access-tree $T_{x}$, it represents as $T_{x}\left(S\right)=1$. $T_{x}\left(S\right)$ outputs 1 if and only if the following conditions are satisfied:

(1) If *x* is a leaf node, $T_{x}\left(S\right)=1$ if and only if att(x)∈S.

(2) If *x* is an interior node, each child $T_{z}\left(S\right)$ of node *x* is individually computed in a recursive way. $T_{x}\left(S\right)=1$ if and only if at least $k_{x}$ children return 1.

## 3. Problem Statement

In this section, we will discuss the system model and definition of our efficient and lightweight fine-grained data sharing scheme for mobile cloud computing.

### 3.1. System Model

**Figure 1:**
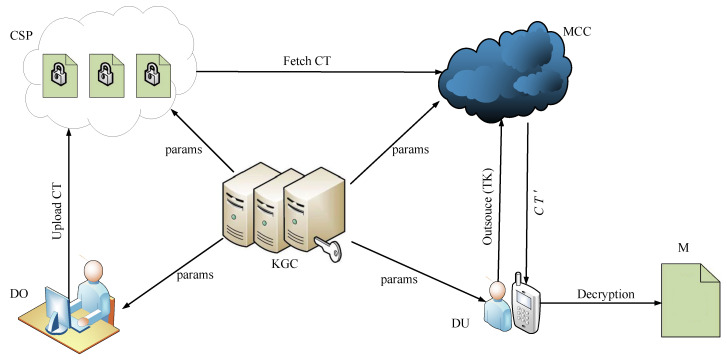
System model of our scheme.

As illustrated in Figure 1, the system framework and interaction among its elements of outsourced ABE scheme for the mobile cloud computing are presented, which consists of five types of entities: Namely, Key Generation Center (KGC), Cloud Service Provider (CSP), Mobile Cloud Computing (MCC), Data Owners (DO) and Data Users (DU).

Briefly speaking, the KGC is responsible for generating and distributing the private and public key pairs for other entities in the system. The CSP is a semi-trusted entity which takes charge of storing the data for DO. DO produces message and encrypts it into ciphertext CT, then uploads it to the CSP for storage or sharing it with others. DO also determines who is permitted to access and decrypt the ciphertext CT. The MCC can seamlessly integrate with the mobile devices and facilitate the mobile devices to process data by virtue of its seemingly infinite storage and powerful computing ability. The DU in this paper mainly refers to the users equipped with the resource-constrained mobile devices, but he/she can efficiently process data with the help of MCC.

### 3.2. Definition of Our Scheme

In this subsection, we provide the detailed definition of our outsourced ABE scheme for the mobile cloud computing, which is composed of the following seven algorithms.

(1) Setup(*k*,*U*). This algorithm is performed by KGC to initialize the system. Taking the security parameter *k* and attribute universe *U* as input, it outputs the public parameters params, and the master secrete key mk.
(2) Extract(params,mk,*S*). This algorithm is performed by KGC. Taking the public parameters params, the master secrete key mk and an attribute set *S* of DU, it extracts the private key for the DU related to the attribute set *S*.
(3) Encrypt(params,*M*,*T*). This algorithm is performed by DO. Taking the public parameters params, the plain message *M*, and access policy *T* as input, it outputs the ciphertext CT.
(4) Decrypt(params,CT,$sk_{S}$, *M*). This algorithm is performed by the DU without using the MCC. Taking the public parameters params, the ciphertext CT and the key set $sk_{S}$ as input, it outputs the message *M*.
(5) $GenTK_{out}$(params,$sk_{S}$,TK). This algorithm is performed by the DU. Taking the public parameters params and the key set $sk_{S}$ as input, it outputs transformation key TK.
(6) $Transform_{out}$(params, CT, TK,$CT^{\prime }$). This algorithm is performed by the MCC. Taking the public parameters params, the ciphertext CT and the transformation key TK, it outputs transformed ciphertext $CT^{\prime }$, which has partially decrypted by the MCC.
(7) $Decrypt_{out}$(params,$CT^{\prime }$,*M*). This algorithm is performed by the DU. Taking the public parameters params and the transformed ciphertext $CT^{\prime }$, it outputs the message *M*.

### 3.3. Security Model

Similar to most existing works, the MCC is assumed as a semi-trust entity, which means that the MCC is an “honest but curious” entity. To be specific, on one hand, the MCC is faithfully performing each operation of the assigned protocol and returns the correct results; on the other hand, the MCC may be curious about the encrypted data contents and may try to learn or infer the sensitive information of the underlying plaintext of the encrypted data by virtue of its powerful computation ability. Based on the security model of Lai et al. [26], in this section, we define a security model for our SLFG-DSS to specify the capabilities and possible actions of the attacker by a game involved two participants: The challenger C and the attacker A. The interactive process between them can be expressed as following steps.

Setup. The attacker A declares a challenging access structure $T^{*}$($T^{*}$ including *l* leaf nodes and the corresponding attributes is $w_{*1},w_{*2},\cdots ,w_{*l}$, respectively). The challenger C runs Setup algorithm with the security parameter *k* and attribute universe *U* to output the master secrete key mk and the system public parameters params, then keeps the master secrete key mk privately and sends the system public parameters params to the attacker A.

Phase 1. The attacker A adaptively issues the following queries:

Key extraction query. The attacker A adaptively chooses an attribute set $S=\{S_{1},S_{2},\cdots ,S_{n}\}$ to launch the private key query; the challenger C returns the corresponding key set $sk_{S}$.

Decryption query. Given a ciphertext $CT_{{}^{*}}$ and an attribute set *S*, the challenger C performs the decryption algorithm Decrypt (CT,$sk_{S}$) and returns the decrypted result *M*.

$Decrypt_{out}$ query. Given certain attribute set *S*, ciphertext CT and $CT^{\prime }$, the challenger C runs $Decrypt_{out}$ algorithm and returns the decryption results *M*.

Challenge. The attacker A submits two messages $M_{0}$, $M_{1}$ with equal length and one access structure $T^{*}$, the challenger C randomly selects b∈{0,1}, then the attacker A returns the challenge ciphertext $CT_{{}^{*}}$ to the attacker A.

Phase 2. The attacker A continues to adaptively initiate the inquiries in phase 1 with the following two restrictions:

(1) A cannot issue the private key query that the selected attribute set satisfy the access structure $T^{*}$.
(2) A cannot make the decryption query over $CT_{{}^{*}}$.

Guess. At the end of the game, the attacker A outputs the guess result $b^{\prime }\in \left\{0,1\right\}$ of *b* and the attacker A succeeds in the game if and only if $b^{\prime }=b$.

The advantage of the attacker A to win the game is defined as:

$$Adv^{IND-SLFG-DSS-CCA2}\left(A\right)=2\mathrm{Pr}\left[b^{{}^{\prime }}=b\right]-1.$$

## 4. Our Concrete Construction

In this section, the concrete construction of our new scheme will be presented in detail as below.

(1) Setup(*k*,*U*). $G_{1},G_{T}$ are two bilinear cyclic groups with order $q\;(\geq 2^{k})$, $e:G_{1}\times G_{1}\to G_{T}$ is a bilinear pair of the two groups, all attributes set is *W*. Lagrange coefficients $L_{i,U}\left(x\right)=\prod_{j\in U,j\neq i}\frac{x-j}{i-j}$, *U* is the number set in $Z_{q}^{}$. Select random number $g,g_{2}$ in $G_{1}$, select $a,\beta \in Z_{q}^{*}$ randomly, calculate $g_{1}=g^{a}$, $g_{3}=g_{2}^{a^{-1}\beta }$; select four anti-collision hash functions $H_{0}:Z_{q}^{*}\to G_{1}$, $H_{1}:G_{T}\to {\;\;\;\{\;\;\;0,1\;\;\;\}\;\;\;}^{k}$, $H_{2}:{\left\{0,1\right\}}^{*}\to G_{1}$ and $H_{3}:G_{T}\to Z_{q}^{*}$. Publish public parameters $params=\{G_{1},G_{T},H_{0},H_{1},H_{2},H_{3},g,g_{1},g_{2},g_{3},e\}$, and keep the master secrete key $mk=\;\;\;\{\;\;\;\alpha ,\beta ,g_{2}^{a}\}$ secretly.

(2) Extract(params,mk,*S*). Given a user’s attribute set $S=\;\;\;\{\;\;\;w_{1},w_{2},\cdots ,w_{n}\}$, the KGC generates a set of keys for the mobile user: $sk_{S}=\left\{\left(g_{2}^{\alpha +d}{\left({g_{1}}^{w_{1}}h_{1}\right)}^{u_{1}},g^{u_{1}}\right),\cdots ,\left(g_{2}^{\alpha +d}{\left({g_{1}}^{w_{n}}h_{n}\right)}^{u_{n}},g^{u_{n}}\right),{g_{1}}^{\beta^{-1}d}\right\}$, where $u_{1},u_{2},\cdots ,u_{n},d$ are n+1 random numbers in $Z_{q}^{*}$, $h_{i}=H_{0}\left(w_{i}\right)\;i=1,2,\cdots ,n$.

(3) Encrypt(params,*M*,*T*). On input message *m*, access tree *T* (including *l* leaf nodes and the corresponding attributes are $w_{*1},w_{*2},\cdots ,w_{*l}$, respectively), return the ciphertext CT by the following steps.

① Select $r\in Z_{q}^{*}$ randomly, and for each node *x*, select a polynomial $q_{x}$ with the degree $d_{x}=k_{x}-1$ in top-down manner, and for root node *R* of the tree, set $q_{R}\left(0\right)=r$. Otherwise, for non-root node *x*, set $q_{x}\left(0\right)=q_{p(x)}\left(index\left(x\right)\right)$, where the p(x) represents the parent node of *x*, index(x) return an unique number associated with the node *x*, which is uniquely assigned to *x* in a certain manner.
② Calculate $h_{*j}=H_{0}\left(w_{*j}\right)\;$, $C_{1}=g_{3}^{r}$, $C_{2}=g^{h}$ and $C_{*j}=\left(C_{*j}^{1},C_{*j}^{2}\right)=\left\{g^{q_{x}\left(0\right)},{\left({g_{1}}^{w_{*j}}h_{*j}\right)}^{q_{x}\left(0\right)}\right\}$ for each leaf node *x*, which is corresponding to a certain attribute in the access tree *T*.
③ Calculate $K=H_{1}\left(e{\left(g_{1},g_{2}\right)}^{r}\right)$, $C_{0}=M⊕K$, $h=H_{3}\left(e{\left(g_{1},g_{2}\right)}^{r},M\right)$.
④ Calculate $\sigma =H_{2}{\left(T,C_{0},C_{1},C_{*1},C_{*2},\cdots ,C_{*l},g^{h}\right)}^{h}$.
⑤ Output $CT=(T,C_{0},C_{1},C_{2},C_{*1},C_{*2},\cdots ,C_{*l},\sigma )$.

(4) Decrypt(params,CT,$sk_{S}$, *M*). Once receiving ciphertext CT, the DU determines whether the attribute set *S* is satisfied with the access structure *T*. If it is not satisfied with the access structure, the DU returns ⊥. Otherwise, the DU can obtain the message *M* by decrypting the ciphertext CT as follows.

① Define a recursive algorithm $Dec(CT,sk_{S},x)$. On input the ciphertext CT, the key set $sk_{S}$ associated with the attribute set *S* and a node *x* in the tree *T*. Denote attr(x) as the true attribute associated with leaf node *x*. The specific decryption process by computing as follows:
  For *x* is the leaf node, the decryption results are returned according to the following two conditions.
  (a) If i=attr(x)∈S, return
    $$\begin{array}{cc} Dec(CT,sk_{S},x)\; & =\frac{e(C_{*x}^{1},g_{2}^{\alpha +d}{\left({g_{1}}^{i}h_{i}\right)}^{u_{i}})}{e(C_{*x}^{2},g^{u_{i}})}\\ \; & =\frac{e(g^{q_{x}\left(0\right)},g_{2}^{\alpha +d}{\left({g_{1}}^{i}h_{i}\right)}^{u_{i}})}{e({\left({g_{1}}^{i}h_{i}\right)}^{q_{x}\left(0\right)},g^{u_{i}})}\\ \; & =e{\left(g_{1},g_{2}\right)}^{q_{x}\left(0\right)}e{\left(g,g_{2}^{d}\right)}^{q_{x}\left(0\right)}. \end{array}$$
  (b) If i=attr(x)∉S, return $Dec(CT,sk_{S},x)=⊥$.
② For *x* is a non-leaf node, if all child nodes *z* of node *x*, the number of nodes which meet $Dec(CT,sk_{S},z)\neq ⊥$ is less than the threshold $k_{x}$, return $Dec(CT,sk_{S},x)=⊥$. Otherwise, randomly select $k_{x}$ child nodes which meet $Dec(CT,sk_{S},z)\neq ⊥$ to form a set ${S_{x}}^{\prime }$, and denote as $S_{x}=\left\{i=index\left(z\right)|z\in S_{x}^{\prime }\right\}$, then proceed as follows.
  $$\begin{array}{cc} Dec(CT,sk_{S},x)\; & =\prod_{z\in {S_{x}}^{\prime }}Dec{\left(CT,sk_{S},z\right)}^{L_{i,S_{x}}\left(0\right)}\\ \; & =e{\left(g_{1},g_{2}\right)}^{q_{x}\left(0\right)}e{\left(g,{g_{2}}^{d}\right)}^{q_{x}\left(0\right)}. \end{array}$$
  (a) Call the $Dec(CT,sk_{S},R)$ algorithm, where *R* is the root node of access tree. We can get $temp^{\prime }=e{\left(g_{1},g_{2}\right)}^{r}e{\left(g,{g_{2}}^{d}\right)}^{r}$, then we can further obtain
    $$temp=\frac{temp^{\prime }}{e(g_{1}^{\beta^{-1}d},g_{3}^{r})}=e{\left(g_{1},g_{2}\right)}^{r}.$$
  (b) Calculate $K=H_{1}\left(temp\right),M=C_{0}⊕K$, $h=H_{3}\left(temp,M\right)$.
  (c) Set $H=H_{2}\left(T,C_{0},C_{1},C_{*1},C_{*2},\cdots ,C_{*l},C_{2}\right)$, if the following two equations $C_{2}=g^{h}$, $e\left(\sigma ,g\right)=e\left(H,C_{2}\right)$ hold, output the message *M*; otherwise, output ⊥.

(5) $GenTK_{out}$(params,$sk_{S}$,TK). The DU can generate the transformation key as follows. He/She firstly selects a random number $t\in Z_{q}^{*}$, and computes the transformation key TK as below.

$$TK=\;\;\;\left\{\;\;\;s{k_{S}}^{\prime },g^{t}\right\}=\left\{\left(g_{2}^{t(\alpha +d)}{\left(g^{w_{1}}h_{1}\right)}^{tu_{1}},g^{tu_{1}}\right),\cdots ,\left(g_{2}^{t(\alpha +d)}{\left(g^{w_{n}}h_{n}\right)}^{tu_{n}},g^{tu_{n}}\right),{g_{1}}^{t\beta^{-1}d},g^{t}\right\}.$$

By adopting the transformation key technique [25], a complicated ciphertext of DO can be transformed to another simple form ciphertext by the MCC acting as a semi-trusted proxy. There exist leakage risks of the decryption key $sk_{S}$ of DU. However, in our scheme, during the transformation key generation procedure, DU adopts the random mask technique through random number *t* to prevent the cloud server and other attacker from obtaining the decryption key $sk_{S}$ of DU. Moreover, *t* is treated as index, thus, the attacker cannot obtain the value of *t* based on the classical DLP problem.

(6) $Transform_{out}$(params, CT, TK,$CT^{\prime }$). This algorithm is performed between the DU and the MCC.

Once receiving the ciphertext CT and transformation key TK, the MCC determines whether the user’s attribute set *S* matches the access structure *T*. If it does not match the access structure, the MCC returns ⊥. Otherwise, the MCC can transform the ciphertext CT by transformation key TK as follows.

① Call $Dec(CT,s{k_{S}}^{\prime },R)$ algorithm to calculate
  $$Tmp_{1}=\frac{Dec(CT,sk_{S}^{\prime },R)}{e(g_{1}^{t\beta^{-1}d},g_{3}^{r})}=e{\left(g_{1},g_{2}\right)}^{tr}.$$
② Calculate $Tmp_{2}=e\left(\sigma ,g^{t}\right),Tmp_{3}=e\left(H,C_{2}\right)$.
③ Return $CT^{\prime }=\left(Tmp_{1},Tmp_{2},Tmp_{3},C_{0},C_{2}\right)$.

The transformed ciphertext $CT^{\prime }$ involve five components, which has partially decrypted by the MCC. To be specific, it contains 3 elements in $G_{T}$, 1 element in $G_{1}$ and k bits random string, thus, the length of the transformed ciphertext $CT^{\prime }$($3\left|G_{T}\right|+\left|G_{1}\right|+k$) is constant, which is independent with the number of the attributes.

(7) $Decrypt_{out}$(params,$CT^{\prime }$,*t*). After received the transformed ciphertext $CT^{\prime }$, the DU calculates $temp=Tmp^{t^{-1}}$, $K=H_{1}\left(temp\right),M=C_{0}⊕K$, $h=H_{3}\left(temp,M\right)$. If the following two equations $C_{2}=g^{h}$ and $Tmp_{2}=Tm{p_{3}}^{t}$ hold, outputs message *M*, otherwise, outputs ⊥.

Apparently, it can be observed that the DU can check the validity of the transformed ciphertext $CT^{\prime }$.

## 5. Security Analysis

Under the security model defined in Section 3.3, in this section, we prove that the proposed SLFG-DSS scheme is secure against the IND-SLFG-DSS-CCA2 with respect to Theorem 1.

**Theorem** **1.** *If the Decisional Bilinear Diffie-Hellman Problem (DBDHP) is difficult in $\left(G_{1},G_{T}\right)$, the proposed scheme is sure against the IND-SLFG-DSS-CCA2 under the random oracle model.*

**Proof.**  Given a random instance $\left(g,g^{a},g^{b},R\right)$, the goal of challenger C is to decide whether *R* is equal to $e{\left(g,g\right)}^{a^{2}b}$.

Setup. At the beginning of the game, the challenger C defines four hash functions and system parameters according to the given instance and returns the results to the adversary A as follows.

$H_{0}\left(w\right)={g_{1}}^{-w}g^{r_{w}}$, where $r_{w}\in Z_{q}^{*}$ is random number.

$H_{1}\left(R\right)=K$, where $K\in {\left\{0,1\right\}}^{k}$ is a random string.

$H_{2}\left(str\right)=X$, where $X\in G_{1}$ is a random number.

$H_{3}\left(R\right)=z$, where $z\in Z_{q}^{*}$ is a random number.

Challenger C randomly selects $r,v,t\in Z_{q}^{*}$, and sends the system parameters $params=(G_{1},G_{T},H_{0},H_{1},H_{2},H_{3},g,g_{1}=g^{a},g_{2}=g^{ar},g_{3}=g^{vr},g^{t},e)$ to the attacker A. The attacker A can only get the hash function value through hash query. Here, the master secrete key $mk=\;\;\{\;\;\;a,\beta =av,g^{a^{2}r}\}$.

Phase 1. The attacker A adaptively launches the following queries:

Key extraction query. The attacker A adaptively selects an attribute set $S=\{S_{1},S_{2},\cdots ,S_{n}\}$ to issue the private key query; the challenger C selects n+1 random numbers: $u_{1},u_{2},\cdots ,u_{n},d^{\prime }$, and returns the corresponding key set

$$\begin{array}{cc} sk_{S} & =\;\;\{\;\;\;(g^{d^{\prime }}{\left({g_{1}}^{w_{1}}h_{1}\right)}^{u_{1}}={g_{2}}^{a+d}{\left({g_{1}}^{w_{1}}h_{1}\right)}^{u_{1}},g^{u_{1}}),\cdots ,(g^{d^{\prime }}{\left({g_{1}}^{w_{n}}h_{n}\right)}^{u_{n}}={g_{2}}^{a+d}{\left({g_{1}}^{w_{n}}h_{n}\right)}^{u_{n}},g^{u_{n}}),g^{v^{-1}d^{\prime }}{g_{1}}^{-v^{-1}}\\ & \;={g_{1}}^{\beta^{-1}d}\;\;\;\}\;\;\;, \end{array}$$

where $h_{i}=H_{0}\left(w_{i}\right)\;i=1,2,\cdots ,n$, $d=d^{\prime }-a$.

Decryption query. Given a ciphertext $CT_{{}^{*}}$, the challenger C firstly performs the key extraction query for attribute set *S* to get $sk_{S}$, then, executes the decryption algorithm Decrypt (CT,$sk_{S}$) and returns the decrypted result *M*.

Note that if the attribute set of the ciphertext satisfies the access tree of the challenged ciphertext, the challenger C just obtains the key set $sk_{S}$ by the key extraction query, but does not return it to the attacker.

$Decrypt_{out}$ query. In this query stage, when the attacker A queries transform information over a certain attribute set *S*, challenger C obtains the key set $sk_{S}$ through the key extraction query, then calculates $s{k_{S}}^{t}$. $TK=\;\;\{\;\;\;({g_{2}}^{ta+td}{\left({g_{1}}^{w_{1}}h_{1}\right)}^{tu_{1}},g^{tu_{1}}),\cdots ,{g_{2}}^{ta+td}{\left({g_{1}}^{w_{n}}h_{n}\right)}^{tu_{n}},g^{tu_{n}}),{g_{1}}^{t\beta^{-1}d}\;\;\;\}\;\;\;$. Once receiving the decryption query over ciphertext $CT^{\prime }$ from the attacker, the challenger C decrypts $CT^{\prime }$ using *t* and returns the decryption results by performing $Decrypt_{out}$ algorithm.

Challenge. The attacker A sends two messages with equal length $M_{0}$, $M_{1}$ and one access structure $T^{*}$($T^{*}$ involves *l* leaf nodes and the corresponding attributes are $w_{*1},w_{*2},\cdots ,w_{*l}$, respectively), the challenger C randomly selects b∈{0,1}, then the attacker A returns the challenge ciphertext $CT_{{}^{*}}$ as follows.

(1) Query hash function $H_{0}\left(w_{*i}\right)\;\;i=1,2,\cdots ,l_{*}$, obtain $r_{w_{*i}}$.
(2) Denote root is the root node of $T^{*}$, set $q_{root}\left(0\right)=r_{*}$. According to the step 1 in encryption algorithm, calculate $q_{x}\left(0\right)$ for each leaf node *x*.
(3) Calculate $C_{*i}=\left\{g^{bq_{x}\left(0\right)},g^{br_{w_{*i}}q_{x}\left(0\right)}\right\}$ for each *x* which is corresponding to an attribute in the access tree $T^{*}$.
(4) Calculate $h=H_{3}\left(R^{br_{*}},m\right)$, $C_{0*}=M⊕H_{1}\left(R^{br_{*}}\right)$, $C_{1*}=g_{3}^{br_{*}}$ and $\sigma_{*}=H_{2}{\left(T^{*},C_{0*},C_{1*},C_{*1},C_{*2},\cdots ,C_{*l_{*}},g^{h}\right)}^{h}$.
(5) Return $CT_{{}^{*}}=\left(T^{*},C_{0*},C_{1*},C_{*1},C_{*2},\cdots ,{C_{*l}}_{{}_{*}},\sigma_{*}\right)$.

Phase 2. The attacker A continues to adaptively initiate the inquiries in phase 1 with the following two restrictions:

(1) A cannot issue the private key query that the selected attribute set satisfy the access structure $T^{*}$.
(2) A cannot make the decryption query over $CT_{{}^{*}}$.

Guess. At the end of the game, the attacker A outputs the guess result $b^{\prime }\in \left\{0,1\right\}$ of *b* and the attacker A succeeds in the game if and only if $b^{\prime }=b$, then challenger C outputs 1, otherwise, outputs 0.

Obviously, $temp_{*}=e\left(g_{2}^{a},g_{}^{br_{*}}\right)$ can be calculated according to the decryption algorithm. Therefore, if $R=e{\left(g,g\right)}^{a^{2}b}$, then $CT_{{}^{*}}$ is the legitimate ciphertext of $m_{b}$, i.e., the challenger C can decide whether *R* is equal to $e{\left(g,g\right)}^{a^{2}b}$ according to the reply of the attacker A.

In addition, there is no failure case during the simulation. Therefore, if the attacker A can broke the scheme with non-negligible probability ε, it means that the challenger C can decide whether *R* is equal to $e{\left(g,g\right)}^{a^{2}b}$ with non-negligible probability. This is a paradox because it is well accepted that the DBDHP problem is intractable. □

## 6. Performance Evaluation

In this section, we first conduct the functionality comparison of our novel scheme with the schemes [14,26,29,45]. Subsequently, we evaluate and compare the efficiency among them from both theoretical analysis and experimental simulation aspects.

### 6.1. Functionality Comparison

In this subsection, we conduct the functionality comparison between our new scheme and several other schemes [14,26,29,45] in terms of the functionalities of outsourcing decryption, verifiability and CCA security. The comparison results are listed in Table 1. As shown in Table 1, it can be observed that scheme [14] is the standardized ABE schemes and cannot support all of the three properties. Scheme [29] can realize outsourcing decryption but not support the properties of verifiability and CCA security. Scheme [26,45] can achieve the security property of outsourcing decryption and verifiability, but fail to support CCA security. Only our proposed novel scheme supports all of the three properties simultaneously, i.e., outsourcing decryption, verifiability, and CCA security.

Based on the above analysis, we could safely draw a conclusion that our novel scheme has better security level than others and can be applicable to the circumstance with higher security level demand.

### 6.2. Performance Analysis

In this subsection, we conduct performance analysis and comparison between our proposed scheme and the other existing scheme [14,26,29,45] from both theoretical analysis and experimental simulation aspects.

Table 2 summarizes the comparison of computation complexity of our novel scheme with the several other schemes [14,26,29,45] which demonstrates the theoretical numerical analysis results.

To facilitate expression, let $T_{pair}$, $T_{G_{1}}$, $T_{G_{T}}$ and $T_{H}$ denote the computational cost of one bilinear pairing operation, one group operation (including exponentiation, multiplication) in $G_{1}$, one group (including exponentiation, multiplication) operation in $G_{T}$, one general one-way hash function operation, respectively.

In Encrypt phase, the DO in reference [14] requires to perform $2N_{1}+1$ group operations in $G_{1}$, 2 group operations in $G_{T}$, and $N_{1}$ general one-way hash function operations, respectively, Therefore, the computation cost is $2T_{T_{G_{T}}}+\left(2N_{1}+1\right)T_{G_{1}}+N_{1}T_{H}$. The DO in reference [29] requires to perform $2+4N_{1}$ group operations in $G_{1}$ and 2 group operations in $G_{T}$, respectively, Therefore, the computation cost is $\left(2+4N_{1}\right)T_{G_{1}}+2T_{G_{T}}$. The DO in reference [26] requires to perform $6+8N_{1}$ group operations in $G_{1}$, 4 group operations in $G_{T}$, and 2 general one-way hash function operations, respectively. Therefore, the computation cost is $\left(6+8N_{1}\right)T_{G_{1}}+4T_{G_{T}}+2T_{H}$. The DO in reference [45] requires to perform $2N_{1}$ group operations in $G_{1}$, and $2+N_{1}$ group operations in $G_{T}$, respectively, Therefore, the computation cost is $2N_{1}T_{G_{1}}+\left(2+N_{1}\right)T_{G_{T}}$. The DO in our proposed scheme requires to perform $4N_{1}+3$ group operations in $G_{1}$, 1 group operations in $G_{T}$, and $N_{1}+3$ general one-way hash function operations, respectively. Therefore, the computation cost is $\left(4N_{1}+3\right)T_{G_{1}}+T_{G_{T}}+\left(N_{1}+3\right)T_{H}$.

In Decrypt phase, the DU in reference [14] needs to execute $2N_{2}+1$ bilinear pairing operations and $2\underset{z\in T}{\Sigma }d_{z}+2$ group operations in $G_{T}$, respectively, Therefore, the computation cost is $\left(2N_{2}+1\right)T_{pair}+\left(2\underset{z\in T}{\Sigma }d_{z}+2\right)T_{G_{T}}$. Reference [29] does not provide the Decrypt algorithm, therefore, the computation cost for Decrypt phase is denoted as None. The DU in reference [26] needs to execute $4N_{2}+2$ bilinear pairing operations, 4 group operations in $G_{1}$, and $4N_{2}+4$ group operations in $G_{T}$, respectively. Therefore, the computation cost is $\left(4N_{2}+2\right)T_{pair}+4T_{G_{1}}+\left(4N_{2}+4\right)T_{G_{T}}$. Reference [45] does not provide the Decrypt algorithm, therefore, the computation cost for Decrypt phase is denoted as None. The DU in our proposed scheme needs to execute $2N_{2}+3$ bilinear pairing operations, 1 group operations in $G_{1}$, $2\underset{z\in T}{\Sigma }d_{z}+1$ group operations in $G_{T}$, and 3 general one-way hash function operations, respectively. Therefore, the computation cost is $\left(2N_{2}+3\right)T_{pair}+T_{G_{1}}+\left(2\underset{z\in T}{\Sigma }d_{z}+1\right)T_{G_{T}}+3T_{H}$.

In Transform phase, reference [14] does not provide the Transform algorithm, therefore, the computation cost for Transform phase is denoted as None. The DU in reference [29] needs to carry out $3N_{2}+2$ bilinear pairing operations, and $4N_{2}+2$ group operations in $G_{T}$, respectively, Therefore, the computation cost is $\left(3N_{2}+2\right)T_{pair}+\left(4N_{2}+2\right)T_{G_{T}}$. The DU in reference [26] needs to carry out $4N_{2}+2$ bilinear pairing operations, and $4N_{2}+2$ group operations in $G_{T}$, respectively, Therefore, the computation cost is $\left(4N_{2}+2\right)T_{pair}+\left(4N_{2}+2\right)T_{G_{T}}$. The DU in reference [45] needs to carry out $4N_{2}$ bilinear pairing operations, $3N_{2}$ group operations in $G_{1}$, $5N_{2}$ group operations in $G_{T}$, and 1 general one-way hash function operations, respectively. Therefore, the computation cost is $4N_{2}T_{pair}+3N_{2}T_{G_{1}}+5N_{2}T_{G_{T}}+1T_{H}$. The DU in our proposed scheme needs to carry out $2N_{2}+3$ bilinear pairing operations, $2\underset{z\in T}{\Sigma }d_{z}+1$ group operations in $G_{T}$, and 1 general one-way hash function operations, respectively. Therefore, the computation cost is $\left(2N_{2}+3\right)T_{pair}+\left(2\underset{z\in T}{\Sigma }d_{z}+1\right)T_{G_{T}}+T_{H}$.

In $Decrypt_{out}$ phase, reference [14] does not provide the $Decrypt_{out}$ algorithm, therefore, the computation cost for $Decrypt_{out}$ phase is denoted as None. The DU in reference [29] has to run 2 group operations in $G_{T}$. Therefore, the computation cost is $2T_{G_{T}}$. The DU in reference [26] has to run 4 group operations in $G_{1}$, 4 group operations in $G_{T}$, and 2 general one-way hash function operations, respectively, Therefore, the computation cost is $4T_{G_{1}}+4T_{G_{T}}+2T_{H}$. The DU in reference [45] has to run 2 group operations in $G_{T}$. Therefore, the computation cost is $2T_{G_{T}}$. The DU in our proposed scheme has to run 1 group operation in $G_{1}$, 2 group operations in $G_{T}$, and 2 general one-way hash function operations, respectively, Therefore, the computation cost is $1T_{G_{1}}+2T_{G_{T}}+2T_{H}$.

Table 2 illustrates that computation complexity for the operation of encryption, decryption and transformation. Here, we mainly focus on the comparison of decryption time. The decryption time of scheme [14] is roughly proportional to the number of attributes, especially with respect to the expensive pairing operations, while it keeps constant in schemes [26,29,45] and ours since they have migrated the heavy operations in the decryption phase to the cloud server and only remain several lightweight operations. However, schemes [29] fails to support the properties of verifiability. Careful observation will further reveal that our scheme slightly outperforms Lai et al.’s scheme [26] because our proposed scheme reduce three element operations in $G_{1}$, two element operations in $G_{T}$, and Fan et al.’s scheme [45] slightly outperforms ours because Fan et al.’s scheme reduce one element operation in $G_{1}$, and two hash operations, however, their scheme is CPA-secure, while ours is CCA2-secure.

**Figure 2:**
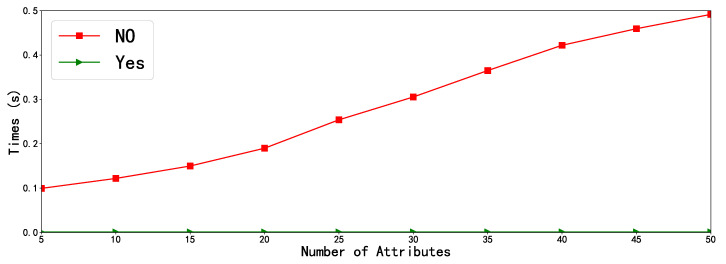
Comparison of outsourcing and non-outsourcing of our scheme, where “Yes” represents our scheme with outsourcing and “No” represents our scheme without outsourcing.

**Figure 3:**
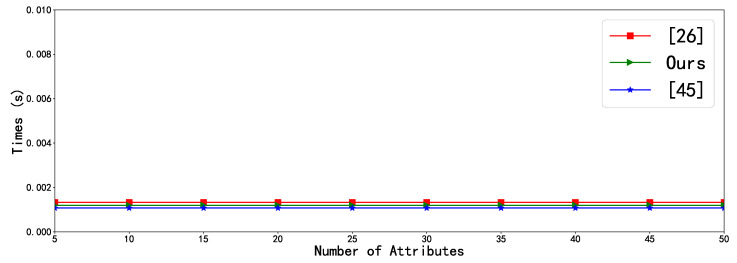
Comparison of outsourced decryption time.

In order to validate theoretical analysis of the efficiency of our proposed scheme, we also conduct the experiment simulation by using a rapidly prototyping development tool Charm 0.43 [46] with Python programming language, and the system platform is Ubuntu 16.04 64-bit operating system with the Intel(R)Core (TM) 4130 CPU @3.40GHz, 4GB RAM. In addition, each experiment simulation is conducted 30 times, and the mean of the experiment results is taken as the final result, which are intuitively illustrated in Figure 2 and Figure 3, respectively.

Figure 2 depicts the performance comparison of outsourced decryption with non-outsourced decryption for our scheme. We can easily find that our scheme with the outsourced decryption method is significantly superior to our scheme without the outsourced decryption method. Apparently, as depicted in Figure 2, the decryption time of the non-outsourced decryption method grows rapidly with the number of attributes, while that of outsourced decryption is nearly constant time at a quite low level. The reason for this we have discussed in previous section.

Figure 3 shows the comparison of outsourced decryption time between our proposal, Lai et al.’s scheme [26], and Fan et al.’s scheme [45]. It is clearly observed that outsourced decryption time of our proposal and Lai et al.’s scheme [26] are roughly similar, but a closer look will find that our scheme slightly outperforms Lai et al.’s scheme [26], which confirms the theoretical analysis discussed in previous subsection. It is also can be seen that Fan et al.’s scheme [45] slightly outperforms than ours, however, their scheme only realizes CPA-security while ours achieves CCA2-security. It is worth and meaningful for mobile uses to enhance the security level from CPA to CCA2 at the very little cost. It can be safely concluded that our proposed novel SLFG-DSS scheme can achieve higher security level and high performance.

## 7. Conclusions

In this paper, we investigate the efficiency bottleneck of using the ABE scheme to achieve fine-grained data sharing in mobile cloud computing and propose a secure and lightweight fine-grained data sharing scheme for mobile cloud computing which simultaneously supports the following desired security properties: (1) Checkability; (2) resisting decryption key exposure; (3) achieving CCA security level. In our novel schemes, by utilizing the transformation key technique, we outsource the most time-consuming pairing operations of the ABE scheme, which previously executed on the mobile device side, and only leaves a slight number of inexpensive operations. The concrete security proof and performance analysis demonstrate that our novel scheme is secure and practical for mobile cloud computing.

Considering the tremendous progress of quantum computers, our future work will be focused on developing some post-quantum-secure outsourced attribute-based encryption schemes from lattice to resist against quantum computer attacks in the near future.

## Figures and Tables

**Table 1:** Comparisons of functionalities.

Scheme	Outsourcing	Verifiability	CCA Security
[14]	No	No	No
[29]	Yes	No	No
[26]	Yes	Yes	No
[45]	Yes	Yes	No
Ours	Yes	Yes	Yes

**Table 2:** Comparison of computation complexity.

Scheme	Encrypt	Decrypt	Transform	$Decrypt_{out}$
[14]	$\left(2N_{1}+1\right)T_{G_{1}}+2T_{T_{G_{T}}}+N_{1}T_{H}$	$\left(2N_{2}+1\right)T_{pair}+\left(2\underset{z\in T}{\Sigma }d_{z}+2\right)T_{G_{T}}$	None	None
[29]	$\left(2+4N_{1}\right)T_{G_{1}}+2T_{G_{T}}$	None	$\left(3N_{2}+2\right)T_{pair}+\left(4N_{2}+2\right)T_{G_{T}}$	$2T_{G_{T}}$
[26]	$\left(6+8N_{1}\right)T_{G_{1}}+4T_{G_{T}}+2T_{H}$	$\left(4N_{2}+2\right)T_{pair}+4T_{G_{1}}+\left(4N_{2}+4\right)T_{G_{T}}$	$\left(4N_{2}+2\right)T_{pair}+\left(4N_{2}+2\right)T_{G_{T}}$	$4T_{G_{1}}+4T_{G_{T}}+2T_{H}$
[45]	$2N_{1}T_{G_{1}}+\left(2+N_{1}\right)T_{G_{T}}$	None	$4N_{2}T_{pair}+3N_{2}T_{G_{1}}+5N_{2}T_{G_{T}}+1T_{H}$	$2T_{G_{T}}$
Ours	$\left(4N_{1}+3\right)T_{G_{1}}+T_{G_{T}}+\left(N_{1}+3\right)T_{H}$	$\left(2N_{2}+3\right)T_{pair}+T_{G_{1}}+\left(2\underset{z\in T}{\Sigma }d_{z}+1\right)T_{G_{T}}+3T_{H}$	$\left(2N_{2}+3\right)T_{pair}+\left(2\underset{z\in T}{\Sigma }d_{z}+1\right)T_{G_{T}}+1T_{H}$	$1T_{G_{1}}+2T_{G_{T}}+2T_{H}$

## Abbreviations

The following abbreviations are used in this manuscript:

ABE	Attribute-Based Encryption
KP-ABE	Key-Policy Attribute-Based Encryption
CP-ABE	Ciphertext-Policy Attribute-Based Encryption
KGC	Key Generation Center
CSP	Cloud Service Provider
MCC	Mobile Cloud Computing
DO	Data Owners
DU	Data Users
TK	Transformation Key
CT	Ciphertext
CPA	Chosen-Plaintext Attack
CCA	Chosen-Ciphertext Attack
PHR	Personal Health Record
DBDH	Decisional Bilinear Diffie-Hellman
PPT	Probabilistic Polynomial Time
